# Supplementary material for: Cystatin M/E ameliorates bone resorption through increasing osteoclastic cell estrogen influx
Source: Res Sq. 2024 May 6:rs.3.rs-4313179. Preprint. [Version 1] doi: 10.21203/rs.3.rs-4313179/v1 (PMC11100902; doi:10.21203/rs.3.rs-4313179/v1)
Supplement: Supplement 1 [file nihpprs4313179v1-supplement-1.pdf]

Supplementary Figures

Supplementary Figure 1

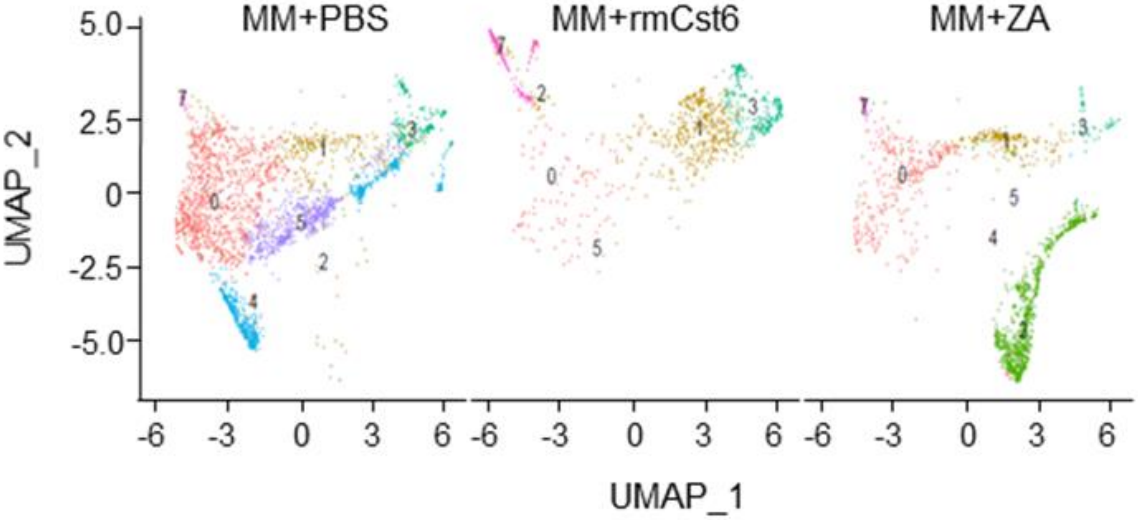

Supplementary Figure 1: UMAP plot of bone marrow macrophages following MM mouse model treatment with either PBS control solution (n = 3), rmCst6 (n = 3), or ZA (n = 2). Compared to control treated mice rmCst6 treatment decreased the amount of M0, M4 and M5 macrophages present in the bone marrow while increasing the percentage of M7 macrophage present. Following ZA treatment; M0, M3, M4 and M5 macrophage sub-clusters are decreased while the M2 macrophage sub-cluster is increased. However, M0 macrophages, which are considered osteoclast precursors are not decreased to the extent of rmCst6 treatment.
